# Dietary modification of blood pressure-lowering effects of antihypertensive drugs – a scoping review

**DOI:** 10.1097/HJH.0000000000004247

**Published:** 2026-02-11

**Authors:** Bristi Miah, Johanna M. Geleijnse, Diederick E. Grobbee, Yvonne T. van der Schouw, Jonathan E. Knikman

**Affiliations:** a Julius Center for Health Sciences & Primary Care, University Medical Center Utrecht, Utrecht University, Utrecht; b Division of Human Nutrition and Health, Wageningen University, Wageningen, The Netherlands

**Keywords:** dietary effect modification of antihypertensive drugs, hypertension, magnesium supplementation, potassium supplementation, sodium restriction

## Abstract

Hypertension remains a major public health concern. Diet is a well established modifiable risk factor for hypertension and may interact with antihypertensive drugs. This scoping review aimed to identify dietary factors that modify the blood pressure (BP) lowering effects of antihypertensive drugs. The review protocol was preregistered on Zenodo and was guided by PRISMA-ScR. Studies were included when participants were treated with a specified antihypertensive drug class and a dietary intervention. Of 7346 screened reports, 43 met inclusion criteria and investigated 16 dietary factors across five antihypertensive drug classes. Evidence for effect modification was available for eight dietary factors in ten drug-diet combinations. Of these, seven combinations suggested modification of the BP lowering effect of antihypertensive drugs, either a trend toward enhancement or attenuation. In addition, sodium restriction, potassium and magnesium supplementation showed consistent BP-lowering effects when added to drug therapy. These findings support the potential of integrating dietary considerations into antihypertensive treatment strategies to improve BP control.

## INTRODUCTION

Hypertension is defined by the World Health Organization (WHO) as a systolic blood pressure (SBP) ≥140 mmHg, a diastolic blood pressure (DBP) ≥90 mmHg, or the use of antihypertensive drugs [1]. Globally, approximately one in three adults aged 30–79 years has hypertension; of which only 54% are formally diagnosed [1,2]. Recent evidence suggests that previous thresholds for defining high BP may have underestimated cardiovascular risk, therefore the American Heart Association and the European Society of Cardiology have recently published more strict definitions, underscoring the importance of BP reduction and control [3,4] Large-scale meta-analyses of randomized controlled trials (RCTs) have indicated that a reduction of 5 mmHg in SBP lowers the risk of major cardiovascular events by approximately 10% [3,4]. This benefit was observed in both normotensive individuals and in patients with established cardiovascular diseases. Given the high prevalence and substantial disease burden of hypertension, optimizing its treatment remains a critical priority.

Several classes of potent antihypertensive drugs are available for the treatment of high BP. First-line drugs include angiotensin-converting enzyme inhibitors (ACEIs), angiotensin II receptor blockers (ARBs), calcium channel blockers (CCBs), and diuretics. These drug classes offer comparable cardiovascular protection, albeit via different mechanisms. ACEIs and ARBs target the renin–angiotensin system to regulate BP, fluid balance, and vascular resistance. CCBs inhibit calcium influx in cardiac and arterial muscle cells, thereby reducing contraction, heart rate, and BP. Diuretics promote sodium and water excretion and prevent fluid retention and BP elevation. Additional options include beta-blockers (BBs), which lower BP by reducing the heart rate and cardiac output. Hypertension treatment typically begins with low dose of two drug classes, with adjustments and escalations as required to achieve BP control [5,6].

Despite the availability of effective antihypertensive drugs, only 21% of patients achieve the guideline-recommended BP targets [1,2]. Poor BP control has been attributed to suboptimal drug selection, reduced drug effectiveness, and poor adherence [7–9]. Adherence challenges often arise from the asymptomatic nature of hypertension, drug side effects, and comorbidities. Interindividual variation in antihypertensive drug response is further shaped by factors such as genetics, sex, hormonal status, age, and dietary factors [10]. However, drug selection in practice is primarily guided by comorbidities and contraindications rather than patient-specific biological or lifestyle characteristics.

Dietary factors represent a particular relevant and underappreciated source of variability because they have the potential to modify drug response through both pharmacokinetic and pharmacodynamic pathways. Acute interactions may occur through concomitant consumption, while habitual dietary patterns may alter drug disposition over time. Patients with comorbidities and suboptimal diets may be particularly susceptible to food-drug interactions [11]. These interactions can affect drug absorption, metabolism, distribution, and excretion. A well known example is the inhibition of the CYP3A4 isoform of the cytochrome P450 (CYP) enzymatic system by grapefruit juice, altering the metabolism and efficacy of certain medications, such as statins [12,13].

Beyond potential pharmacokinetic interactions, diet warrants attention as it is a key risk factor for hypertension, and dietary consumption varies on an individual basis [14]. It is unclear whether incorporating an individual's dietary habits when choosing an antihypertensive drug class could enhance treatment response, as the impact of diet on antihypertensive drug effectiveness is underexplored. This scoping review aimed to identify dietary factors, including dietary regimens, supplements, and specific food components that modify the BP-lowering effects of antihypertensive drugs.

## METHODS

### Protocol and reporting guidelines

The protocol for this scoping review was developed and made publicly available via Zenodo [15]. The review was guided by the Preferred Reporting Items for Systematic Reviews and Meta-Analyses extension for Scoping Reviews (PRISMA-ScR) [16]. Data supporting the findings are available from the corresponding author upon reasonable request.

### Search strategy

A systematic search was conducted in PubMed on July 8, 2025, covering relevant studies since the inception of the database. The search terms included synonyms for “food intake” and dietary factors, as well as “antihypertensive drugs” in titles and abstracts. Filters were applied based on predefined inclusion criteria for the study design. A detailed search string can be found in Supplementary Item 1.

### Study selection

Study inclusion criteria for study design were RCTs and longitudinal observational studies, that is, case-cohort, nested case-control, and cohort studies in participants aged 18 years or older who received antihypertensive drugs, excluding those studies in which participants received the drug primarily for comorbidities. Other inclusion criteria were that studies had to specify both the antihypertensive drug class and dietary factors under investigation, and report the mean BP values at baseline and after intervention. No exclusion criterium was applied to single or multiple dose interventions, as both can provide relevant information on acute BP responses and on potential dietary influences on drug absorption or metabolism. Because this review focused on any modification of the drug-BP response rather than solely on long-term BP control, both types of studies were eligible. Reports on medically specialized diets were excluded. A minor deviation from the protocol was made, excluding studies reporting only pharmacokinetic outcomes as they did not directly address the research question. Reports in English or Dutch were eligible. The Covidence software was used for study selection and data extraction [17].

### Quality appraisal

Although formal risk-of-bias assessment is not required for scoping reviews, we conducted an exploratory appraisal of study quality to support interpretability of the findings. We appraised five categories of risk of bias for each included study: bias arising from crossover allocation or randomization, bias due to deviations from intended intervention, bias due to missing outcome data, bias in measurement of the outcome, bias in selection of the reported result. For each category of bias we assigned either low, moderate or high risk of bias. We then assigned overall risk of bias based on the appraisal of the five categories of bias by assigning an overall low risk of bias if all categories were assigned low risk, a moderate risk if at least one category was appraised moderate risk and none was appraised high risk, and we assigned an overall high risk of risk if at least one category was assigned high risk.

### Assessment of the modifying effects by dietary factors

**FIGURE 1:**
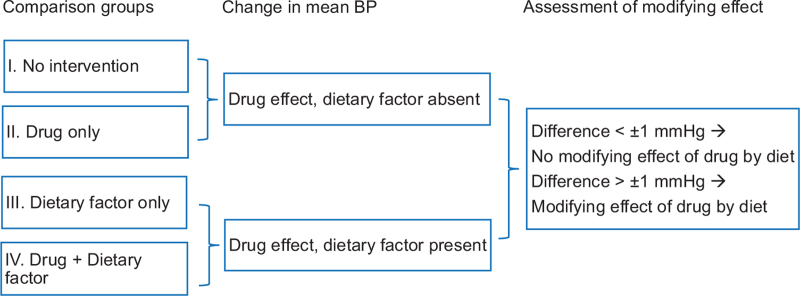
Comparison of blood pressure changes between the four groups to differentiate between synergistic and additive effects of dietary factors.

To investigate whether and to what extent dietary factors modify the BP lowering effects of antihypertensive drugs, we compared BP across four comparison groups (Fig. 1).

1. No intervention
2. Antihypertensive drug only
3. Dietary factor only
4. Combined intervention: Antihypertensive drug and dietary factor

The absolute effect on BP of a dietary factor was defined as the difference in BP (ΔBP) between group IV and group II.

To evaluate potential effect modification by dietary factors, we compared the drug-induced change in BP in the absence of dietary factors (ΔBP: II – I) with the drug-induced change in the presence of dietary factors (ΔBP: IV – III). The difference between these two effects, calculated as (IV – III) – (II – I), was used to quantify the presence and direction of effect modification. A dietary factor was considered to modify the antihypertensive effect if this difference exceeded ±1 mmHg. Because no validated or clinically established thresholds exist for defining effect modification in the context of dietary influences on antihypertensive drug response, and the dietary exposure is expected to exert smaller BP effects than pharmacological treatments, we applied a minimal descriptive cut-off of ±1 mmHg to indicate potential directional trends. This threshold was used solely for exploratory purposes and does not imply clinical significance. If one or more of the comparisons groups were missing, separate comparisons were conducted based on the available groups. The results were summarized in forest plots to explore potential trends in BP response.

### Calculation of mean differences and confidence intervals

Mean BP values for each comparison group were extracted from the original study reports. Where available, the mean difference in BP between groups was also extracted; otherwise, it was calculated by subtracting the BP value for the reference group from that of the treatment group. To calculate the confidence intervals (CIs) of the mean difference, different formulas were applied for paired and unpaired data. For both instances, an intermediate calculation was performed for the standard error (SE) of the BP difference via the standard deviation (SD). For paired data, the variance was estimated with the formula:


$$\mathrm{var}iance=SD^{2}\;group_{1}\;+\;SD^{2}\;group_{2}-2r\;*SDgroup_{1}*SDgroup_{2}$$

where *r* represents the correlation coefficient between the two mean BP measurements. Although the correlation coefficient may vary across studies, a value of 0.6 was assumed, in line with previous literature [18,19]. The SE of the BP difference was calculated as:

SE*=√*(variance/*n*),

where n is the sample size. For unpaired data, the SE was calculated as:

SE*= √*((SD^2^ group_1_/*n*_1_) + (SD^2^ group_2_/*n*_*2*_)).

Subsequently, the CI was calculated for both paired and unpaired data as:

CI = mean difference in BP ± *t*_α/2_ * SE.

The *t*_α/2_ stands for the critical *t*-value, which was determined based on a 95% confidence level and degrees of freedom of the sample sizes. The resulting mean differences in BP, with the corresponding CIs, were visualized in forest plots. These plots were used both to explore trends in studies with limited comparison groups and to illustrate the magnitude and direction of blood pressure effects by antihypertensive drug class and dietary factor across all available studies. CIs for mean BP differences could not be calculated if the SD or SE of the mean BP values was not reported. In such cases, a CI of zero was assigned solely to enable the presentation of the mean BP difference data points in forest plots.

Further, calculation of the CI for the difference of the drug effects in the presence and absence of dietary factors ((IV – III) – (II – I)) was done similarly to the calculation described above. In this case, the variance and SDs of IV – III and II – I were first calculated with the same formulas above and then the SE for the difference of the drug effects was calculated. In short, SD^2^ group in the formulas was substituted by the SD^2^ II – I and SD^2^ IV – III.

## RESULTS

### Study characteristics

**FIGURE 2:**
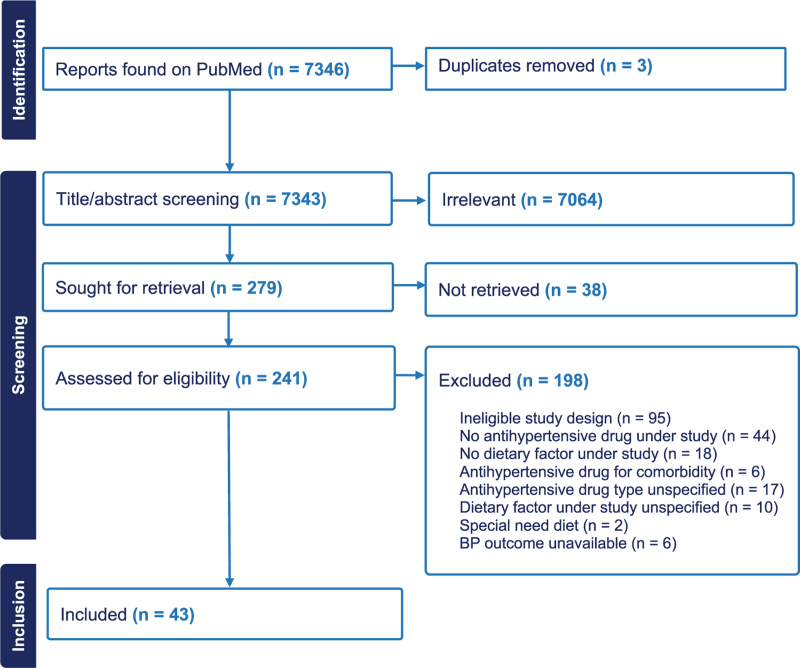
Study selection flowchart.

Of 7346 reports screened, 43 studies met the inclusion criteria. These studies investigated 16 dietary factors across five classes of antihypertensive drugs, as outlined in the PRISMA flowchart (Fig. 2). Four studies assessed multiple dietary factors [20–23], and eight examined more than one antihypertensive drug class [24–31].

The most frequently examined dietary factor was restricted sodium intake, reported in 19 studies [23,24,29,31–47]. Potassium supplementation was examined in five studies [21,23,46–48], and two studies investigated a combination of high potassium and low sodium intake [20,25]. Magnesium supplementation was assessed in three studies [49–51], and a combination of potassium and magnesium supplementation was reported in two studies [21,23]. Furthermore, the Dietary Approaches to Stop Hypertension (DASH) diet was evaluated in two studies [20,52], while sesame oil was examined in three studies [22,53,54]. Vitamin D supplementation was reported in two studies [60,61]. In addition, several dietary factors were assessed in single studies, including calcium supplementation [62], coffee intake [63], barberry supplementation [27], grapefruit juice intake [59], linoleic acid supplementation [65], *Balsemodendron mukul* (*B. mukul*) supplementation [66], a diet high in fruits and vegetables [67], and groundnut and sunflower oil intake (Table 1, Supplemental Digital Content) [22]. Of the included studies, 21 were appraised as having an overall low risk of bias and 22 studies as having a moderate risk of bias. No study was classified as having a high risk of bias.

When stratified by antihypertensive drug class, ACEIs were investigated in 16 studies, followed by ARBs in ten studies, CCBs in 13 studies, BBs in three and diuretics in 11 studies. Regarding study design, 22 studies employed a crossover design, 18 studies used an RCT design, and an additional three used nonrandomized experimental designs. The studies were conducted across 21 countries, including eight in the United States, and six in the Netherlands. Population size varied from 11 to 787 and duration of intervention varied from a single dose to six months. A comprehensive summary of all included studies, first categorized by dietary factors and subsequently by antihypertensive drug class, is presented in Table 1.

**TABLE 1:** Overview of main study characteristics of the 43 included studies

Study	Drug class	Drug	Dietary factor	Study design	Study intervention protocol	Study population	Risk of bias assessment
**Sodium**							
Kocks *et al.* 2005	ACEI	Enalapril	Sodium; high, low	R crossover	2 weeks enalapril/placebo; - 1 week 200 mmol/day sodium - 1 week 50 mmol/day sodium	17 healthy men	Moderate
Weir *et al.* 1998	ACEI; CCB	Enalapril; isradipine	Sodium; high, low	RCT	3 weeks of either drug with low sodium or high sodium	397 hypertensives	Moderate
Navis *et al.* 1987	ACEI	Enalapril	Sodium; high, low	Crossover	16 days of 10 mg twice daily enalapril - 8 days 200 mmol/day sodium - 8 days 50 mmol/day sodium	9 hypertensives	Moderate
Chrysant *et al.* 2000	ACEI; CCB	Enalapril, isradipine	Sodium; high, low	RCT	- drug or placebo for 4 weeks + 50–80 mmol/day sodium - drug or placebo for 4 weeks + 200–250 mmol/day high sodium	236 hypertensives with salt sensitivity	Moderate
Resnick *et al.* 1994	CCB	Nifedipine	Sodium; high, low	R crossover	2 months of nifedipine - 1 month 200 mmol/day sodium - 1 month 50 mmol/day sodium	19 hypertensives	Moderate
McCarron *et al.* 1997	CCB	Isradipine	Sodium; high, low	R crossover	4 weeks each - sodium restriction and isradipine - placebo and 100 mmol sodium	99 hypertensives	Moderate
Leonetti *et al.* 1987	CCB	Nifedipine	Sodium; high, low	Crossover	- 6–8 days 20 mmol sodium + single dose nifedipine - 6–8 days 100 mmol sodium + single dose nifedipine	11 hypertensives	Low
Gu *et al.* 2016	ARB	Fimasartan	Sodium; high, low	R crossover	- 7 days 50 mmol/day + single dose filmasartan - 7 days 300 mmol/day + single dose filmasartan	16 healthy men	Moderate
Vogt *et al.* 2008	ARB	Losartan	Sodium; high, low	R crossover	- 6 weeks 50 mmol/day sodium+ losartan - 6 weeks 200 mmol/day sodium+ losartan - 6 weeks 50 mmol/day sodium+ placebo - 6 weeks 200 mmol/day sodium+ placebo	34 proteinuric patients	Low
MacGregor *et al.* 1987	ACEI	Captopril	Sodium; low	R crossover	- 2 weeks captopril+ low sodium - 2 weeks captopril+ high sodium	15 hypertensives	Low
Humalda *et al.* 2015	ACEI	Lisinopril	Sodium; low	R crossover	- 3 weeks lisinopril+ low sodium - 3 weeks lisinopril+ high sodium	52 CKD patients with residual proteinuria	Low
Azizi *et al.* 1995	ACEI; ARB	Captopril; losartan	Sodium; low	R crossover	- single dose of drug + low sodium - placebo + low sodium	12 normotensive men	Low
Kimura *et al.* 1988	ACEI; CCB; BB	Captopril; nicardipine; propanolol	Sodium; low	RCT	- 1 week low sodium+drug (per drug) - 1 week regular sodium+drug	24 hypertensives	Low
Agnoli *et al.* 1999	ACEI	Enalapril	Sodium; low	RCT	- 6 weeks sodium depletion+ single dose drug	14 hospitalized women without renal, CVD and metabolic disease	Low
Mills *et al.* 1993	ACEI	Captopril	Sodium; low	Crossover	25 days hospital stay 10 mmol/day sodium and twice daily captopril or placebo	12 hypertensive men	Low
Houlihan *et al.* 2002	ARB	Losartan	Sodium; low	Crossover	8 weeks either low or high sodium - 4 weeks drugs - 4 weeks placebo	21 hypertensives with elevated albumin excretion rate	Low
Erwteman *et al.* 1984	Diuretic; BB	Chlorthalidone; metoprolol	Sodium; low	R crossover	8 weeks either low 70 mmol/day or regular sodium - 4 weeks drugs - 4 weeks placebo	94 hypertensives	Moderate
Morgan *et al.* 1988	CCB	Nifedipine	Sodium; low	Crossover	Sodium restriction - 2 weeks nifedipine - 2 weeks placebo	8 hypertensives	Low
Parijs *et al.* 1973	Diuretic	Sprinolactone; hydrochlorothiazide	Sodium; low	R crossover	4 periods of 4 weeks; crossover of low or regular sodium and diuretic or placebo	18 hypertensives	Moderate
**Other dietary factors**							
Kirpizids *et al.* 2005	ARB	Candesartan	DASH	RCT	16 weeks of either candesartan only or candesartan with DASH diet	201 hypertensives	Moderate
Huggins *et al.* 2011	ARB or ACEI		DASH-like; high K low Na;	R crossover	4-week crossover diets DASH like and low Na high K.	94	Low
Langford *et al.* 1991	BB; Diu	Atenolol; chlorthalidone	high K low Na; weight loss	RCT	- 6 months drug + diet	787 clinic patients	Low
Kaplan *et al.* 1985	Diuretic		K	R crossover	6 weeks KCl 60 mmol/day or placebo tablets with diuretic	16 hypertensives	Moderate
Lumme *et al.* 1989	Diuretic	Hydrochlorothiazide	K; K and Mg	R crossover	- 8 weeks potassium as hydrochloride 1 g twice daily - 8 weeks drug 50 mg daily + placebo - 8 weeks drug 50 mg daily +combination of potassium hydrochloride 1 g and magnesium hydroxide 500 mg twice daily	11 mild hypertensives with ventricular extrasystoles	Low
Zhang *et al.* 2018	Diuretic	Indapamide	KCl	RCT	- 1.25-.250 mg/day inpapamide + placebo - 1.25–2.50 mg/day inpapamide +KCl 40 mmol/day	92 hypertensives	Moderate
Malta *et al.* 2016	ARB or ACEI		K rich diet	RCT	- 4 weeks high K diet of fruits and vegetables+ drug - 4 weeks usual diet+ drug	20 hypertensives	Low
Vongpatanasin *et al.* 2023	Diuretic	Chlorthalidone	K; K and Mg	Blinded RCT	- 16 weeks drug+ KCl or - 16 weeks drug+ KMgCit	69 hypertensives	Low
Cunha *et al.* 2017	Diuretic	Hydrochlorothiazide	Mg	RCT	- 6 months 600 mg twice daily magnesium chelate + drug - 6 months placebo + drug	35 hypertensive women	Moderate
Dyckner *et al.* 1988	Diuretic		Mg	RCT	- 6 months of 15 mmol/day of magnesium aspartate hydrochloride +drug - 6 months placebo + drug	39 ambulatory patients	Moderate
Taylor *et al.* 1988	Diuretic	Indapamide	Mg	Blinded RCT	- 8 weeks 6× per day 535 mg magnesium+ 2.5 mg/day indapamide - 8 weeks 6× per day 535 mg magnesium+ placebo	27 hypertensives	Moderate
Guerrero-Romero *et al.* 2018	ACEI	Captopril	Mg	RCT	- 4 months receiving 2.5 g of MgCl_2_ +drug - 4 months placebo +drug	79 outpatients	Moderate
Sato *et al.* 1998	CCB	Manidipine	Ca	CT	3 days 8 g/day salt and 600 mg/day calcium + single 20 mg oral manidipine	30 hypertensives	Low
Bernini *et al.* 2013	ARB	Telmisartan	vit D	CT	Single dose 300 000 IU cholecalciferol at time 0+ 80 mg/day telmisartan from day -15 to week 8	18 hypertensive inpatients	Moderate
Bislev *et al.* 2018	ARB	Valsartan	vit D	RCT	2 weeks valsartan 80 mg/day intake with either 70 ug/day vit D3 intake or placebo	41 postmenopausal women between age 60–79 age	Low
Sankar *et al.* 2006	BB;Diu	Atenolol; hydrochlorothiazide	Sesame oil	Crossover	use of sesame oil as only edible oil for 45 days and then withdrawal for 45 days + drug	50 hypertensives	Moderate
Devarajan *et al.* 2016	CCB	Nifedipine	Sesame oil blend	RCT	- sesame oil blend for 60 days - nifedipine 20 mg/day for 60 days - sesame oil blend + nifedipine 20 mg/day for 60 days.	300 hypertensives	Moderate
Sankar *et al.* 2005	CCB	Nifedipine	Sesame oil; groundnut oil; sunflower oil	CT	- 60 days per oil+drug/placebo	530 hypertensives	Moderate
Christensen *et al.* 2002	CCB	Dilitazem	Grapefruit	R crossover	12 mg single dose diltiazem with alternately 250 ml grapefruit juice or water on two study days with 13–38 days in between	10 healthy men	Low
Goraya *et al.* 2012	ACEI		NaHCO_3_; fruit & vegetables	RCT	30 days of either no intervention, sodium biocarbonate or fruits and vegetables +drug	120 CKD 1 and CKD 2 patients	Low
Bailey *et al.* 2016	CCB	Felodipine	Coffee	R crossover	acute 3-way randomised crossover: subjects got either 1. black coffee, 2. water + felodipine or 3 black coffee +felodipine	13 healthy people	Low
Emamat *et al.* 2022	all		Barberry	RCT	10 g/day dried purple-black barberry powder or placebo for 2 months + drug	78 hypertensives	Moderate
Panneerselvam *et al.* 2005	CCB	Nifedipine	*B. mukul*	Blinded RCT	either nifedipine 10 mg/day, *B. mukul* 1.5 g/day or both for 6 weeks	57 hypertensives	Low
Zhao *et al.* 2009	ACEI	Ramipril	Conjugated linoleic acid	Blinded RCT	2.5 g/day conjugated linoleic acid and 37.5 mg/day ramipril or placebo with ramipril	80 obese hypertensives	Moderate

ACEI, angiotensin converting enzyme-inhibitor; ARB, angiotensin receptor blocker; BB, beta blocker; CCB, calcium channel blocker; mmol/d, mmol per day; RCT, randomized controlled trial.

### Dietary factors modifying the effect of antihypertensive drugs

Effect modification by dietary factors was assessed across ten unique combinations of dietary factors and antihypertensive drug classes, as identified in nine studies (Table 2). No combination was evaluated in more than one study, preventing formal statistical assessment. Among these, seven dietary factors demonstrated evidence of modifying the BP-lowering effect, while three showed no modifying effect. In four combinations, the dietary factor showed a trend toward an enhanced antihypertensive response: sodium restriction combined with a BB (−4.2 mmHg), a DASH-like diet with an ACEI/ARB (−1.8 mmHg), *B. mukul* supplementation with a CCB (−4.7 mmHg), and a low-sodium/high-potassium diet with a diuretic (−2.9 mmHg). In contrast, a trend toward attenuation of the BP-lowering effect of drugs was observed for three combinations: magnesium supplementation with a diuretic, vitamin D with an ARB, and sodium restriction with an ARB, resulting in increases of 5.0, 9.8 and 2.4 mmHg, respectively. Further, from the CIs of the difference of the drug effects all but one contained the arbitrary cutoff point ±1 mmHg, which might indicate the differences are not statistically significant. Nonetheless, a trend of effect modification is shown.

**TABLE 2:** Effect of modification by dietary factors

Study	Dietary factor	Drug class	Difference in drug effect [95% CI]	Effect modification trend
Erwteman 1984	Sodium restriction	BB	−4.2 [−6.4, −2.0]	Enhancement
Erwteman 1984	Sodium restriction	Diu	+0.2 [−2.3, 4.5]	Neutral
Houlihan 2002	Sodium restriction	ARB	+2.4 [−5.8, 10,6]	Attenuation
Taylor 1988	Magnesium	Diu	+5.0 [−9.7, 19.7]	Attenuation
Huggins 2011	DASH-like	ARB/ACEI	−1.8 [−6.6, 3.0]	Enhancement
Sato 1998	Calcium	CCB	−1.0 [−10.7, 8.7]	Neutral
Devarajan 2016	Sesame oil	CCB	0.0 [−4.1, 4.1]	Neutral
Panneerselvam 2005	*B. mukul*	CCB	−4.7 [−14.5, 5.1]	Enhancement
Bislev 2018	Vitamin D	ARB	+9.8 [NA]	Attenuation
Langford 1991	Low sodium/high potassium diet	Diu	−2.9 [NA]	Enhancement

ACEI, angiotensin converting enzyme-inhibitor; ARB, angiotensin receptor blocker; BB, beta blocker; CCB, calcium channel blocker.

In addition, eight combinations demonstrated an absolute benefit, defined as the difference in BP between antihypertensive monotherapy (group II) and adding a dietary factor (group VI), ranging from −21.0 mmHg to −0.9 mmHg (Table 2). In contrast, no additional benefit was observed when calcium was added to a CCB regimen (0 mmHg), while vitamin D supplementation in combination with an ARB was associated with a rise in BP (+4.2 mmHg).

### Effect of adding dietary interventions to antihypertensive therapy

**FIGURE 3:**
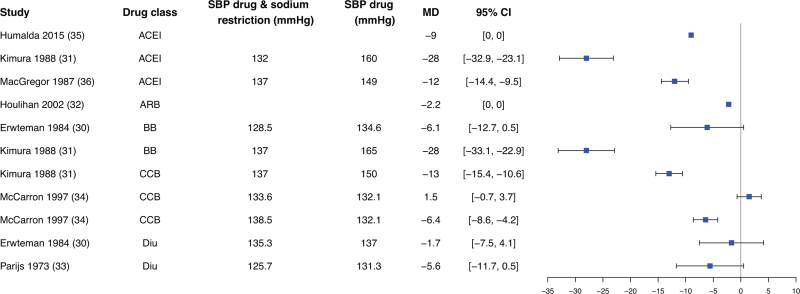
Mean difference in blood pressure change between intake of combination of low sodium with antihypertensive drug versus antihypertensive drug only. 95% CI, 95% confidence interval; BP, blood pressure; MD, mean difference; mmHg, millimetres of mercury.

**FIGURE 4:**
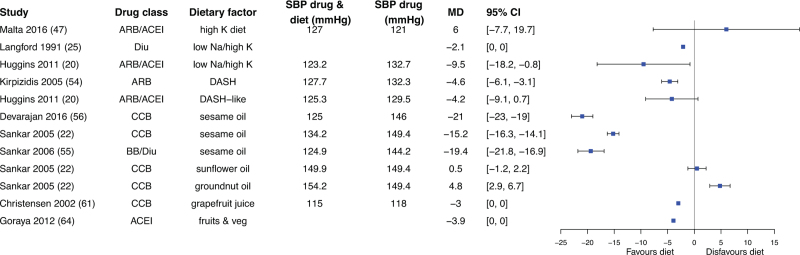
Mean difference in blood pressure change between intake of combination of diet factor with antihypertensive drug versus antihypertensive drug only. 95% CI, 95% confidence interval; BP, blood pressure; MD, mean difference; mmHg, millimetres of mercury.

**FIGURE 5:**
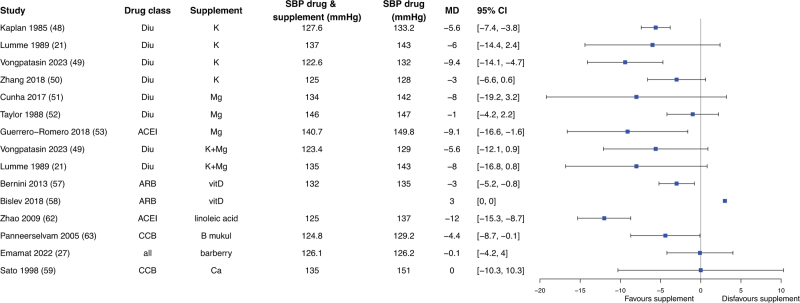
Mean difference in blood pressure change between intake of combination of supplemental diet factor with antihypertensive drug versus antihypertensive drug only. 95% CI, 95% confidence interval; BP, blood pressure; MD, mean difference.

In some studies, data of the dietary factor alone was missing, these studies could not be used to assess true effect modification. However, they did allow estimation of the absolute effect of adding a dietary factor to antihypertensive treatment, defined as the difference in mean BP between the combination of antihypertensive therapy and dietary factor versus antihypertensive drug treatment alone (Fig. 1). Given the large number of studies focusing on sodium, these findings are presented separately in Fig. 3. Effects of other dietary factors consumed as part of a diet are described in Fig. 4, and effects of supplemental dietary factors in Fig. 5.

Most dietary factors were associated with an absolute reduction of the mean BP across all five antihypertensive drug classes. Sodium restriction consistently decreased mean BP beyond the effect of antihypertensive treatment, with observed changes ranging from -28 mmHg to +1.5 mmHg (Fig. 3) [30–33,35,36,63]. This effect was consistent across all drug classes. Of the 11 sodium-related interventions, statistical testing was reported for 10, with six showing statistically significant differences [30–32,35,36].

Other dietary factors consumed as part of diet also demonstrated additional BP-lowering effects, with changes ranging from −21 mmHg to +6 mmHg (Fig. 4) [20,22,25,46,52–54,62]. Sesame oil, in combination with either CCBs or BBs in combination with diuretics, significantly reduced BP in three studies [22,53,54]. In contrast, sunflower and groundnut oils did not yield statistically significant effects [22].

The DASH diet combined with ACEIs or ARBs was assessed in two studies of which one reported a statistically significant BP reduction [52], while the other did not perform statistical testing [20]. Similarly, two interventions combining a high-potassium diet intervention with ACEIs/ARBs showed no significant BP reduction [46], or lacked statistical testing [20]. In contrast, a high-potassium/low-sodium diet with diuretics led to a significant BP reduction [25], as did a diet rich in fruits and vegetables with ACEIs [62]. Grapefruit juice combined with CCBs did not result in additional BP-lowering effects [59].

Most dietary factors consumed through supplementation were associated with additional reductions in BP, with changes ranging from −12 mmHg to +3 mmHg (Fig. 5) [20,21,23,27,47,51,56,60,61,64]. Potassium and magnesium supplementation, predominantly studied with diuretics, were evaluated in nine interventions. Five reported statistically significant reductions in BP [21,47,49,51], three showed no significant effect [23,50], and one was not subjected to statistical testing [64]. Two studies assessed vitamin D supplementation in combination with ARBs, neither demonstrating a statistically significant additional BP-lowering effects [55,56]. Among other supplements, linoleic acid and barberry were each associated with a significantly reduced BP [27,60], whereas calcium supplementation showed no effect [57], and the study investigating *B. mukul*[61] did not conduct statistical testing.

### Effect of adding antihypertensive drugs to dietary interventions

In contrast to the previous section, this analysis focused on the additional BP lowering effect of initiating antihypertensive therapy in individuals adhering to a dietary intervention. Findings are summarized in Figures 1–4, Supplemental Digital Content. These figures present the results for restricted and regular or high sodium intake, other dietary factors consumed by food, and those administered as supplements. Across all dietary factors, antihypertensive drugs consistently produced an additional reduction in mean BP beyond the effect of the dietary factor alone. The magnitude of this effect varied substantially, but the direction was uniform across dietary types.

Following a sodium-restricted diet, the addition of antihypertensive drugs resulted in greater BP reductions compared with sodium restriction alone, with observed changes ranging from −17 to 0 mmHg (Figure 1, Supplemental Digital Content) [24,26,28,30,32, 38,43–45,65–68]. Although ACEIs and ARBs were the most frequently studied drug classes, the BP lowering response to sodium restriction was similar across drug classes. Of the 17 interventions, 13 reported statistically significant reductions in BP[24,26,28,30,32,38,42,65–68], three showed no significant effect [30,43,44] and one study did not conduct statistical testing for BP outcomes [45].

Several studies have also assessed the influence of regular or high sodium intake compared with restricted sodium intake, typically representing a difference of approximately 150 mmol/day. In these studies, BP reductions were more pronounced when baseline BP was elevated due to regular or high sodium intake compared with sodium restriction, particularly in combination with CCBs. BP changes ranged from −28 mmHg to −6.8 mmHg (Figure 2, Supplemental Digital Content) [24,38,42,43,66–68]. Of the nine interventions, six demonstrated statistically significant reductions in BP [32,38,41,66,68], two showed no significant effects [24,67] and one study did not conduct statistical testing for BP [43].

The effects of adding antihypertensive drugs to other dietary factors are summarized in Figures 3 and 4, Supplemental Digital Content. For supplemental dietary factors, BP changes ranged from −16 mmHg to 0 mmHg, with no substantial differences across drug classes (Figure 3, Supplemental Digital Content) [50,56,57,61,69]. Only the intervention with calcium supplementation demonstrated a statistically significant BP reduction [57]. Similarly, the addition of antihypertensive drugs to dietary interventions such as the DASH-like diet yielded further BP reductions ranging from −18 mmHg to −0.1 mmHg (Figure 4, Supplemental Digital Content). Conversely, a BP increase of 4 mmHg was observed when antihypertensive therapy was combined with coffee consumption [20,25,54,58]. None of these interventions were tested for statistical significance.

## DISCUSSION

This scoping review identified multiple dietary factors that appear to modify the BP-lowering effects of antihypertensive drugs. While factors such as sodium restriction and DASH-like dietary patterns are well established in lowering BP, their potential to modify drug effects has been less well characterized. Notably, possible modifying effects were also observed for less expected factors, including magnesium, calcium, and vitamin D. These modifying trends were observed in both directions, either enhancing or attenuating the pharmacological response. Additionally, several dietary factors such as sodium restriction, potassium or magnesium supplementation, and substitution with sesame oil were associated with further BP reductions when used in combination with antihypertensive drugs. These findings suggest that dietary factors may either enhance or attenuate the BP-lowering effect of antihypertensive drugs, depending on the specific drug-diet combination.

Although the data were insufficient for formal statistical testing, the observed trend toward enhancement of the BP-lowering effects of BBs by sodium restriction, and its trend toward attenuation when combined with ARBs, suggests that the modifying effects by dietary factors depend on drug class. Caution is warranted in interpreting modifying effects of magnesium, calcium, sesame oil, *B. mukul*, vitamin D, and low sodium in combination with high potassium, as these findings were derived from single studies. Further, the CIs of the difference also require cautious interpretation. Nevertheless, these findings highlight the need for further research into how dietary interventions influence antihypertensive drug response. Such research holds promise for individualizing treatment by identifying specific dietary factors that amplify the BP-lowering effect of antihypertensive drugs, thereby improving overall BP control.

Sodium restriction emerged as the most extensively studied dietary factor in this review, consistently demonstrating additional BP-lowering effects across all classes of antihypertensive drugs. Evidence suggests that limiting sodium intake could substantially improve BP reduction, regardless of the drug class. Proposed mechanisms underlying these BP-lowering effects include reduced water retention, decreased peripheral vascular resistance, improved endothelial function, and modulation of sympathetic activity [70]. Despite the consistent direction of the effect, the magnitude of BP reduction varied widely across studies (ranging from −28 mmHg to +1.5 mmHg), likely reflecting heterogeneity in baseline sodium intake, the degree of sodium restriction (typically 50–100 mmol/day), population characteristics (e.g., hypertensive status, sex, and age), and the intensity of dietary guidance provided [71,72]. Notably, more pronounced BP reductions were observed in studies involving ACEIs, which may reflect the greater number of studies, rather than an inherently greater effect [70]. However, evidence suggests sodium restriction may particularly enhance the antihypertensive effect of renin–angiotensin–aldosterone system (RAAS) blockade. Reduced sodium intake influences local tissue RAAS activity in the kidney, vasculature and the brain as well as the immune system, thereby augmenting the BP-lowering effects of ACEIs [73]. Although sodium restriction appears to be universally beneficial, its effects may be particularly pronounced in individuals with salt-sensitive hypertension [74]. The 2024 ESC guidelines accordingly emphasize sodium reduction as an important nonpharmacological measure to reduce BP [75]. This highlights the potential for targeted dietary recommendations to enhance the effectiveness of antihypertensive therapy [76].

Beyond sodium restriction, other dietary factors such as potassium and magnesium may offer further therapeutic benefit. BP reduction appears to be more pronounced when diuretics are combined with potassium or magnesium supplementation. The BP-lowering effects of potassium and magnesium supplementation may result from increased sodium excretion and regulation of the renin-angiotensin-aldosterone system [77]. Potassium, in particular, inactivates the sodium chloride co-transporter, thereby reducing renal sodium reabsorption [78]. Diuretics, while effective, are known to affect potassium and magnesium homeostasis, potentially leading to hypokalaemia [79,80]. However, potassium and magnesium supplementation or the use of potassium-sparing diuretics can mitigate these effects, although they may also lead to hyperkalaemia without proper monitoring. These findings underscore the importance of potassium and magnesium in hypertension management, suggesting that adequate potassium (3500 mg/day) and magnesium (300–350 mg/day) intake may contribute to clinical BP reduction [81,82]. Moreover, the 2024 ESC guidelines explicitly recommended achieving adequate dietary potassium intake as part of nonpharmacological measures for BP reduction [75].

In addition to micronutrients, other dietary factors have been investigated for their potential to improve BP-lowering effects of antihypertensive drugs. Sesame oil, substituted for other edible oils in patients receiving CCBs and BBs/diuretics, demonstrated substantial BP lowering effects [22,53,54]. These interventions ranged from 45–60 days. While it remains unclear whether the observed BP reductions are attributable to sesame oil itself or whether the oil it replaces contributes to BP elevation, postulated mechanisms include the antioxidant effects of lignans such as sesamin and sesamol, vitamin E, and unsaturated fatty acids present in sesame oil [53]. It is well established that saturated and trans fatty acids promote atherosclerosis and may contribute to hypertension, whereas mono- and polyunsaturated fatty acids can attenuate atherosclerosis [83]. However, the specific antihypertensive properties of sesame oil have not been confirmed. The large BP reductions of up to 21 mmHg within the relatively short intervention periods observed in these studies warrant cautious interpretation [22,53,54]. These findings are preliminary, and further well designed, rigorous trials are necessary before clinical recommendations can be confidently made.

Other dietary factors, such as vitamin D, calcium, barberry, and linoleic acid supplementation, were assessed less frequently, but demonstrated BP-lowering effects when combined with antihypertensive drugs [27,55,57,60]. Furthermore, the DASH diet, specifically designed for BP management, also showed additional benefit when implemented alongside antihypertensive drug treatment. These findings support further exploration into how comprehensive dietary patterns may enhance antihypertensive treatment response. Grapefruit juice was also identified as a dietary factor that may augment the BP-lowering response of CCBs. This interaction is likely mediated by inhibition of CYP3A4, which increases the systemic exposure of CCBs, such as felodipine [84,85]. However, this may also heighten the risk of side effects, emphasizing the need for caution when consuming grapefruit juice with prescribed CCBs [86]. Although several of these dietary factors show promise in enhancing the effects of antihypertensive drugs, the limited number of studies and their methodological heterogeneity underscore the need for well designed trials to validate these findings and elucidate the underlying mechanisms.

### Strengths and limitations

This scoping review addresses an underexplored yet potentially clinically relevant topic by systematically examining the interplay between dietary factors and antihypertensive drug effects. To our knowledge, this is the first review systematically studying the influence of dietary factors on the BP lowering effect of antihypertensive drugs. Key strengths include the comprehensive identification of distinct dietary factors associated with BP reduction, and the distinction between modifying and absolute effects. The study was guided by PRISMA-ScR, enhancing methodological transparency and reproducibility [18]. In addition, an optional and exploratory risk of bias assessment was performed to improve interpretability of the included studies.

Nevertheless, several limitations warrant consideration. First, the extensive range of potential dietary interactions means that some relevant dietary factors may have been inadvertently overlooked. Second, substantial heterogeneity across studies, including study design, sample size, duration of the intervention, outcome definition, and statistical reporting, limited comparability. A meta-analytic approach would have required comparability in both dietary exposure and the antihypertensive drug class across studies. For the effect modification analyses, no two studies examined the same dietary exposure-drug combination, preventing pooling. For the analyses evaluating the addition of a dietary factor to antihypertensive therapy or the addition of antihypertensive therapy to a dietary intervention, a limited number of studies met this matching requirement. However, these studies differed markedly in design and analytical approach, complicating interpretation and pooling of results. The direction and magnitude of reported effects were therefore aggregated and summarized in Table 2 and visualized using forest plots. Third, most modifying effects were based on single studies, limiting the robustness and generalizability of these findings. Fourth, assumptions regarding correlation coefficients and CIs during data synthesis may introduce uncertainty. Finally, publication bias cannot be excluded, as studies reporting null or unfavourable dietary effects may have not been published.

### Implications

The findings of this review suggest that commonly consumed dietary factors may influence the effectiveness of antihypertensive drug treatment, highlighting a potential opportunity to improve hypertension management. Given the high global prevalence and societal burden of uncontrolled hypertension, as well as the clinical relevance of even modest BP reductions, further research into the interplay between dietary factors and antihypertensive drugs is warranted.

Although most included studies were controlled dietary interventions, their findings raise the questions whether in real-world settings, similar interactions may occur through habitual intake of certain foods or supplements. For example, dietary sodium restriction or potassium supplementation could enhance treatment response in specific drug classes, while others such as grapefruit juice or high magnesium may attenuate it. These potential interactions underscore the importance of understanding how dietary patterns interact with pharmacological therapy to guide optimal, and potentially personalized, antihypertensive treatment strategies. In clinical practice, physicians may consider reinforcing ESC guideline-based dietary recommendations, particularly sodium restriction and adequate potassium intake, when initiating or adjusting antihypertensive therapy. Although the evidence summarised here is exploratory, these dietary measures may enhance BP-lowering response and achievement of treatment targets.

## CONCLUSION

This scoping review identified several dietary factors that appeared to modify the BP-lowering effects of certain drug classes, either enhancing or attenuating treatment response. While these findings are often based on single studies, they underscore the need for further research to confirm these interactions and elucidate their underlying mechanisms. A better understanding of these drug-diet effects could inform more effective, and potentially tailored strategies for BP control, and ultimately cardiovascular risk reduction.

## ACKNOWLEDGEMENTS

The authors gratefully acknowledge the statistician Peter Zuithoff who contributed to the calculations described in the Methods section.

Funding: Funded by the European Union. Views and opinions expressed are however those of the author(s) only and do not necessarily reflect those of the European Union or the European Health and Digital Executive Agency (HaDEA). Neither the European Union nor the granting authority can be held responsible for them.

This work is also supported by the UKRI Horizon Europe Guarantee scheme.

Part of this work was presented as a poster presentation at the WEON 2025 Conference.

### Conflicts of interest

There are no conflicts of interest.

## Supplementary Material

Supplemental Digital Content
