# Supplementary material for: Dietary modification of blood pressure-lowering effects of antihypertensive drugs – a scoping review
Source: J Hypertens. 2026 Feb 11;44(5):717–30. doi: 10.1097/HJH.0000000000004247 (PMC13034768; doi:10.1097/HJH.0000000000004247)
Supplement: Supplemental Digital Content [file jhype-44-717-s001.docx]

# Supplementary:

###### Supplementary Item 1. Full search query

((food*[Title/Abstract] OR diet*[Title/Abstract] OR nutri*[Title/Abstract] OR beverage*[Title/Abstract] OR drink*[Title/Abstract] OR consum*[Title/Abstract] OR intake[Title/Abstract] OR ingest* [Title/Abstract] OR digest*[Title/Abstract] OR snack*[Title/Abstract] OR phosph*[Title/Abstract] OR coffee*[Title/Abstract] OR caffeine [Title/Abstract] OR tea[Title/Abstract] OR supplement*[Title/Abstract] OR dairy*[Title/Abstract] OR milk [Title/Abstract] OR yoghurt [Title/Abstract] OR yogurt[Title/Abstract] OR vitamin [Title/Abstract] OR cholecalciferol*[Title/Abstract] OR mineral[Title/Abstract] OR minerals[Title/Abstract] OR potassium*[Title/Abstract] OR sodium* [Title/Abstract] OR salt*[Title/Abstract] OR oil [Title/Abstract] OR “omega 3” [Title/Abstract] OR botanical*[Title/Abstract] OR grapefruit*[Title/Abstract] OR eat[Title/Abstract] OR eating[Title/Abstract] OR licorice[Title/Abstract] OR liquorice[Title/Abstract] OR St. John’s Wort[Title/Abstract] OR magnesium[Title/Abstract] OR alcohol*[Title/Abstract] OR ethanol [Title/Abstract] OR iron[Title/Abstract] OR ferrous fumarate[Title/Abstract] OR star coin[Title/Abstract] OR Panax quinquefolius[Title/Abstract] OR Allium sativum[Title/Abstract] OR garlic[Title/Abstract] OR Salvia miltiorrhiza[Title/Abstract] OR Silybum marianum[Title/Abstract] OR Curcuma longa[Title/Abstract] OR turmeric[Title/Abstract] OR Echinacea[Title/Abstract] OR Ginkgo biloba[Title/Abstract] OR Hypericum perforatum[Title/Abstract] OR Camellia sinensis[Title/Abstract] OR valerian* [Title/Abstract] OR cranberr* [Title/Abstract] OR kava[Title/Abstract] OR danshen[Title/Abstract] OR sage [Title/Abstract] OR ginseng[Title/Abstract] OR “milk thistle” [Title/Abstract] OR broccoli[Title/Abstract] OR sprout*[Title/Abstract] OR cabbage*[Title/Abstract] OR fiber*[Title/Abstract] OR fibre*[Title/Abstract] OR “black cohosh”[Title/Abstract] OR actaea racemosa [Title/Abstract] OR saw palmetto [Title/Abstract] OR goldenseal[Title/Abstract] OR leafy[Title/Abstract] OR cheese*[Title/Abstract] OR chocolate*[Title/Abstract] OR yohimbine[Title/Abstract] OR hawthorne[Title/Abstract] OR roselle[Title/Abstract] OR guggul[Title/Abstract] OR piperine[Title/Abstract] OR capsicum[Title/Abstract] OR cannabidiol*[Title/Abstract] OR “folic acid”[Title/Abstract] OR gelatin[Title/Abstract] OR hemp*[Title/Abstract] OR hibiscus[Title/Abstract] OR probiotic*[Title/Abstract] OR zinc[Title/Abstract] OR cocoa[Title/Abstract] OR polyphenol[Title/Abstract] OR flavonoid*[Title/Abstract] OR isoflavone*[Title/Abstract] OR lignan[Title/Abstract] OR soy[Title/Abstract] OR phytoestrogen*[Title/Abstract] OR nutraceutical* [Title/Abstract] OR herb* [Title/Abstract] OR berr* [Title/Abstract] OR wine* [Title/Abstract] OR beer* [Title/Abstract])

AND ("Antihypertensive Agents" [Mesh] OR “antihypertensive agent*”[Title/Abstract] OR anti-hypertensive*[Title/Abstract] OR antihypertensive*[Title/Abstract] OR "hypertension drug" [Title/Abstract] OR "hypertension drugs" [Title/Abstract] OR "blood pressure drug" [Title/Abstract] OR "blood pressure drugs" [Title/Abstract] OR “blood pressure medication” [Title/Abstract] “beta blocker”[Title/Abstract] OR “beta blockers” [Title/Abstract] OR “alpha blocker” [Title/Abstract] OR “alpha blockers” [Title/Abstract] OR diuretic*[Title/Abstract] OR ACE[Title/Abstract] OR angiotensi*[Title/Abstract] OR ARB[Title/Abstract] OR calcium channel[Title/Abstract] OR vasodilator*[Title/Abstract] OR "mineralocorticoid receptor antagonist*”[Title/Abstract])

Filters applied: Clinical Study, Clinical Trial, Clinical Trial, Phase I, Clinical Trial, Phase II, Clinical Trial, Phase III, Clinical Trial, Phase IV, Comparative Study, Controlled Clinical Trial, Multicenter Study, Observational Study, Randomized Controlled Trial, Twin Study, Humans, Dutch, English.

###### Supplementary Table 1: Number of studies reporting on a dietary factor

| Dietary factor | Number of studies |
| --- | --- |
| Sodium restriction/ supplementation | 19 |
| Potassium supplementation | 5 |
| High potassium/low sodium diet | 2 |
| Magnesium supplementation | 3 |
| Potassium/magnesium combination supplementation | 2 |
| DASH diet | 2 |
| Sesame oil as substitution oil | 3 |
| Vitamin D supplementation | 2 |
| Calcium supplementation | 1 |
| Coffee intake | 1 |
| Barberry supplementation | 1 |
| Grapefruit juice intake | 1 |
| Linoleic acid supplementation | 1 |
| *B. mukul* | 1 |
| Diet high in fruits and vegetables | 1 |
| Groundnut oil as substitution oil | 1 |
| Sunflower oil as substitution oil | 1 |

###### Supplementary Table 2: Effect modification by dietary factors (elaborate version). ACEI, angiotensin converting enzyme-inhibitor; ARB, angiotensin receptor blocker; CCB, calcium channel blocker; BB, beta blocker

| Study | Dietary factor | Drug class | Mean SBP (mmHg) | | | | Delta SBP (mmHg) | | | | Effect modification trend |
| --- | --- | --- | --- | --- | --- | --- | --- | --- | --- | --- | --- |
|  |  |  | No Intervention | Drug alone | Dietary factor alone | Drug + Dietary factor | Absolute effect of diet | Drug effect: diet absent | Drug effect: diet present | Difference in drug effect [95% CI] |  |
|  |  |  | I | II | III | IV | IV - II | II - I | IV - III | (IV - III) - (II - I) |  |
| Erwteman 1984 | Sodium restriction | BB | 142.9 | 134.6 | 141.0 | 128.5 | -6.1 | -8.3 | -12.5 | -4.2 [−6.4, -2.0] | Enhancement |
| Erwteman 1984 | Sodium restriction | Diu | 142.9 | 137.0 | 141.0 | 135.3 | -1.7 | -5.9 | -5.7 | +0.2 [-2.3, 4.5] | Neutral |
| Houlihan 2002 | Sodium restriction | ARB | 109.3 | 104.6 | 104.7 | 102.4 | -2.2 | -4.7 | -2.3 | +2.4 [-5.8, 10,6] | Attenuation |
| Taylor 1988 | Magnesium | Diu | 157.0 | 147.0 | 151.0 | 146.0 | -1.0 | -10.0 | -5.0 | +5.0 [-9.7, 19.7] | Attenuation |
| Huggins 2011 | DASH-like | ARB/ACEI | 127.8 | 129.5 | 125.4 | 125.3 | -4.2 | +1.7 | -0.1 | -1.8 [−6.6, 3.0] | Enhancement |
| Sato 1998 | Calcium | CCB | 150.0 | 135.0 | 151.0 | 135.0 | 0.0 | -15.0 | -16.0 | -1.0 [-10.7, 8.7] | Neutral |
| Devarajan 2016 | Sesame oil | CCB | 164.0 | 146.0 | 143.0 | 125.0 | -21.0 | -18.0 | -18.0 | 0.0 [−4.1, 4.1] | Neutral |
| Panneerselvam 2005 | *B. mukul* | CCB | 125.3 | 129.2 | 125.6 | 124.8 | -4.4 | +3.9 | -0.8 | -4.7 [−14.5, 5.1] | Enhancement |
| Bislev 2018 | Vitamin D | ARB | 137.2 | 127.4 | 131.6 | 131.6 | +4.2 | -9.8 | 0 | +9.8 [NA] | Attenuation |
| Langford 1991 | Low sodium/ high potassium diet | Diu | 134.2 | 124.5 | 136.2 | 123.6 | -0.9 | -9.7 | -12.6 | -2.9 [NA] | Enhancement |

######

###### Supplementary Figure 1: Mean difference in blood pressure change between intake of combination of low sodium with antihypertensive drug versus low sodium only. MD, Mean difference; BP, blood pressure; 95% CI, 95% confidence interval; mmHg, millimetres of mercury.

######

###### Supplementary Figure 2: Mean difference in blood pressure change between intake of combination of high sodium with antihypertensive drug versus high sodium only. MD, Mean difference; BP, blood pressure; 95% CI, 95% confidence interval.

######

###### Supplementary Figure 3: Mean difference in blood pressure change between intake of combination of supplemental diet factor with antihypertensive drug versus supplemental diet factor only. MD= Mean difference; BP=blood pressure; 95% CI, 95% confidence interval; mmHg, millimetres of mercury.

######

Supplementary Figure 4: Mean difference in blood pressure change between intake of combination of diet factor with antihypertensive drug versus diet factor only. MD= Mean difference; BP=blood pressure; 95% CI, 95% confidence interval; mmHg, millimetres of mercury. ^not statistically tested; `mean arterial pressure.
